# Psychometric Evaluation of the Impulsive-Compulsive Behaviours Checklist (ICB) in a Spanish Prison Population

**DOI:** 10.3390/ejihpe15090187

**Published:** 2025-09-18

**Authors:** Francisca López-Torrecillas, Isabel Ramírez-Uclés, F. Pablo Holgado-Tello, Lucas Muñoz-López

**Affiliations:** 1 Center Research Mind Brain and Behavior (CIMCYC), University of Granada, Campus de Cartuja s/n, 18071 Granada, Spain; fcalopez@ugr.es (F.L.-T.); lucasml@ugr.es (L.M.-L.); 2 Department of Personality, Assessment and Psychological Treatments, Universidad Nacional de Educación a Distancia (UNED), Juan del Rosal, 10, 28040 Madrid, Spain; 3 Department of Behavioral Sciences Methodology, Universidad Nacional de Educación a Distancia (UNED), Juan del Rosal, 10, 28040 Madrid, Spain; pfholgado@psi.uned.es

**Keywords:** impulsivity, compulsivity, psychometrics, prison population, scale validation

## Abstract

Impulsivity and compulsivity are key transdiagnostic constructs implicated in addictive and criminal behaviors, often overlapping under the broader concept of behavioral dysregulation. While impulsivity has been widely assessed using experimental tasks and self-report measures, few tools specifically target compulsivity in forensic populations. This study aimed to adapt and validate the Spanish version of the Impulsive-Compulsive Behaviours Checklist (ICB) in a prison sample. The ICB was administered to 700 incarcerated men (mean age = 37.33 years) following a rigorous translation and back-translation procedure, along with pilot testing for clarity. Exploratory and confirmatory factor analyses revealed a stable two-factor structure consistent with theoretical expectations. Internal consistency was satisfactory (McDonald’s omega and Cronbach’s alpha = 0.79–0.80), and convergent validity was supported by significant correlations with the UPPS-P Impulsive Behavior Scale, the Obsessive Beliefs Questionnaire (OBQ-44), the European Addiction Severity Index (EuropASI), and the Symptom Checklist-90-Revised (SCL-90-R). These findings support the Spanish ICB as a valid and reliable tool for assessing impulsive and compulsive traits in forensic contexts. Its use may enhance the identification of differentiated clinical profiles and inform targeted interventions for mental health and reintegration in prison populations.

## 1. Introduction

Impulsivity and compulsivity are transdiagnostic constructs implicated in a broad spectrum of psychiatric disorders and maladaptive behaviors. Although traditionally studied as independent dimensions, growing evidence suggests they frequently co-occur and interact, particularly in clinical and forensic populations (López-Torrecillas, 2025; Robbins et al., 2024). Impulsivity is commonly defined as a predisposition toward rapid, unplanned reactions to internal or external stimuli without regard for negative consequences (American Psychiatric Association, 2013). Compulsivity, in contrast, refers to rigid, repetitive, and maladaptive behaviors aimed at reducing internal distress or avoiding perceived threats, often without achieving any real satisfaction or reward (Obsessive Compulsive Cognitions Working Group [OCCWG], 2005).

Neuropsychological models have associated impulsivity with hypoactivity in prefrontal regions, whereas compulsivity has been linked to hyperconnectivity in cortico-striatal circuits (Cong et al., 2024; Overmeyer & Endrass, 2025). These neural patterns are especially relevant in disorders involving impaired self-regulation, such as substance use disorders, obsessive–compulsive disorder, and antisocial personality disorder. Incarcerated populations show disproportionately high rates of such conditions, underscoring the importance of accurate and context-sensitive assessment tools (Brassard & Joyal, 2022; Clarke et al., 2024).

However, most existing measures assess impulsivity and compulsivity separately and often lack ecological validity in forensic settings. Instruments such as the UPPS-P Impulsive Behavior Scale (Verdejo-García et al., 2010) and the Obsessive Beliefs Questionnaire-44 (OBQ-44; Obsessive Compulsive Cognitions Working Group [OCCWG], 2005) primarily focus on cognitive or emotional traits, potentially overlooking the diversity of problematic behaviors typically observed in prison environments (Van Timmeren et al., 2018). This highlights the need for dimensional, behaviorally grounded tools that assess both constructs jointly.

The Impulsive-Compulsive Behaviours Checklist (ICB; Guo et al., 2017) was developed to address this gap. This 34-item self-report measure evaluates a broad range of maladaptive behaviors classified as predominantly impulsive or compulsive, offering a transdiagnostic and behavior-focused approach. While the ICB has shown clinical utility in diverse populations, no validated Spanish version currently exists for incarcerated individuals. Given the critical role of impulsivity and compulsivity in criminal behavior, functional impairment, and recidivism, culturally adapted and psychometrically sound tools are urgently needed in forensic contexts.

Therefore, the present study aimed to adapt the ICB into Spanish and validate its use in a male prison population. Specifically, we examined its factorial structure, internal consistency, and convergent validity using widely accepted clinical and forensic measures.

## 2. Methodology

### 2.1. Participants

A total of 900 incarcerated men from the Granada Penitentiary Centre (Spain) were approached using a stratified probabilistic sampling method, based on age, nationality, and type of offense to ensure representativeness of the prison population. Out of those approached, 700 individuals voluntarily agreed to participate and met the inclusion criteria. The final sample had a mean age of 37.33 years (SD = 9.09). Inclusion criteria were: (a) age between 18 and 55 years, to control for age-related neurocognitive changes and ensure comparability with previous validation studies; (b) absence of severe physical illnesses or psychiatric conditions (e.g., schizophrenia or major depressive disorder); and (c) not receiving psychopharmacological treatment at the time of evaluation. While the exclusion of physical illnesses might seem restrictive, certain medical conditions (e.g., neurological disorders) can significantly affect cognitive performance and behavior, potentially confounding results.

Each participant underwent a one-on-one clinical interview to confirm eligibility. The complete assessment battery was administered individually. The total sample was randomly divided into two independent subsamples: Sample A (n = 413) was used for exploratory factor analysis (EFA), and Sample B (n = 278) for confirmatory factor analysis (CFA). Randomization was conducted using SPSS random sampling procedures, ensuring balanced demographic and clinical characteristics across subsamples. Sociodemographic and criminal background characteristics are summarized in Table 1.

### 2.2. Instruments

#### 2.2.1. Demographic, Criminal, and Institutional Behavior Interview

A structured interview was developed specifically for this study to collect demographic data, criminal history, and prison-related information, following Spanish correctional regulations (Royal Decree, 1201/1981). It included items on educational level, marital status, nationality, type of offense, and duration of incarceration.

#### 2.2.2. Impulsive-Compulsive Behaviours Checklist (ICB)

The ICB (Guo et al., 2017) is a 34-item self-report instrument designed to assess problematic behaviors categorized along a continuum of impulsivity and compulsivity. Items reflect behaviors such as aggression, gambling, substance use, excessive cleaning, and compulsive checking. Each item is rated on a 4-point Likert scale (1 = “never” to 4 = “always”), with an optional checkbox indicating whether the behavior causes distress. The original scale demonstrated strong psychometric properties in clinical samples. For the purposes of this study, we refer to the two emerging factors as Impulsive Behaviors (Factor 1) and Compulsive Behaviors (Factor 2), to enhance clarity and avoid confusion.

#### 2.2.3. UPPS-P Impulsive Behavior Scale

The UPPS-P (Verdejo-García et al., 2010) assesses five dimensions of impulsivity: (1) Negative Urgency (tendency to act impulsively under distress), (2) Lack of Premeditation (difficulty considering consequences before acting), (3) Lack of Perseverance (difficulty maintaining focus on tasks), (4) Sensation Seeking (preference for excitement), and (5) Positive Urgency (impulsivity in positive emotional states). The Spanish version has shown adequate internal consistency (α = 0.59–0.82) and good factorial validity in both clinical and community populations.

#### 2.2.4. Obsessive Beliefs Questionnaire-44 (OBQ-44)

The OBQ-44 (Obsessive Compulsive Cognitions Working Group [OCCWG], 2005; Nogueira-Arjona et al., 2012) assesses dysfunctional beliefs associated with obsessive–compulsive disorder across three subscales: (1) Responsibility/Threat Estimation, (2) Perfectionism/Certainty, and (3) Importance/Control of Thoughts. Respondents rate each item on a 7-point Likert scale (1 = “disagree very much” to 7 = “agree very much”). The Spanish adaptation has demonstrated excellent internal consistency (total α = 0.95; subscales α = 0.85–0.89) and test–retest reliability.

#### 2.2.5. European Addiction Severity Index (EuropASI)

The EuropASI (Bobes et al., 1996) is a semi-structured interview used widely in clinical and forensic contexts to assess substance use severity across seven domains: Medical, Employment/Support, Alcohol Use, Drug Use, Legal Status, Family/Social Relationships, and Psychological Health. Due to the homogeneity of our sample in terms of legal status (all participants were incarcerated), this domain was excluded. Severity ratings range from 0 (no problem) to 9 (extreme problem), and the instrument has demonstrated good validity and reliability in Spanish samples.

#### 2.2.6. Symptom Checklist-90-Revised (SCL-90-R)

The SCL-90-R (Derogatis & Savitz, 2002; González de Rivera et al., 2002) is a 90-item self-report measure of psychological distress. It assesses nine symptom dimensions: (1) Somatization, (2) Obsessive–Compulsiveness, (3) Interpersonal Sensitivity, (4) Depression, (5) Anxiety, (6) Hostility, (7) Phobic Anxiety, (8) Paranoid Ideation, and (9) Psychoticism. It also provides three global indices: Global Severity Index (GSI), Positive Symptom Total (PST), and Positive Symptom Distress Index (PSDI). The Spanish version has been validated in both clinical and prison populations with good internal consistency (α > 0.70).

### 2.3. Procedure

The original Impulsive-Compulsive Behaviours Checklist (ICB) was translated into Spanish using a standardized parallel back-translation method (Brislin, 1986), in accordance with cross-cultural adaptation guidelines (Castellet et al., 2014; Núñez et al., 2005). First, a bilingual clinical psychologist translated the instrument into Spanish. A second bilingual professional, blinded to the original, independently translated the Spanish version back into English. This process was repeated by two additional translators. An expert panel—comprising the four translators and two clinical psychology researchers—evaluated semantic, conceptual, and functional equivalence, reaching consensus on a final version that retained the format and instructions of the original.

To assess clarity and comprehension, the Spanish version was pilot-tested with two small samples (n = 30 each): university students and members of the general public in Granada. Participants provided written feedback on item clarity and format. Based on this, minor adjustments were made to improve linguistic precision and acceptability.

Four trained researchers administered the entire assessment battery (structured interview, ICB, and psychological instruments) individually in dedicated rooms within the prison. Participants received verbal and written information about the study and signed informed consent. All assessments were anonymous and confidential. The study protocol was approved by the Research Ethics Committee of the University of Granada (code: 396/CEI/2018) and adhered to the Declaration of Helsinki.

### 2.4. Statistical Analyses

Descriptive statistics (means, standard deviations, skewness, and kurtosis) were calculated for all ICB items. Analyses were conducted using SPSS 25 and FACTOR 12.03.02 software.

To assess internal structure, both exploratory factor analysis (EFA) and confirmatory factor analysis (CFA) were performed. EFA was conducted on Sample A (n = 413) to explore the latent structure beyond the original model proposed by Guo et al. (2017), given that the initial CFA did not yield adequate model fit. CFA was then conducted on Sample B (n = 278) to confirm the factorial structure derived from the EFA. Due to the ordinal nature of the ICB Likert-scale items, analyses were based on polychoric correlation matrices and robust unweighted least squares (RULS) estimation (Holgado-Tello et al., 2010).

Model fit was evaluated using multiple indices: Satorra–Bentler χ^2^, Root Mean Square Error of Approximation (RMSEA), Comparative Fit Index (CFI), Non-Normed Fit Index (NNFI), and Standardized Root Mean Square Residual (SRMR). Following conventional guidelines, CFI and NNFI ≥ 0.95 and RMSEA ≤ 0.06 indicate good fit, while RMSEA values between 0.06 and 0.08 are considered acceptable (Hu & Bentler, 1999; MacCallum et al., 1996; McDonald & Ho, 2002).

Internal consistency was assessed using McDonald’s omega (ω) and Cronbach’s alpha (α), with values ≥ 0.70 considered acceptable (Ventura-León & Caycho-Rodríguez, 2017). Corrected item-total correlations were also calculated.

Convergent validity was examined using Spearman correlations between ICB factor scores and subscales of the UPPS-P, OBQ-44, EuropASI, and SCL-90-R. The Legal Status subscale from EuropASI was excluded due to sample homogeneity (all participants were incarcerated). Statistical significance was set at *p* < 0.005 (two-tailed).

## 3. Results

### 3.1. Descriptive Item Analysis

Table 2 displays the descriptive statistics for the 34 items of the Impulsive-Compulsive Behaviours Checklist (ICB), including means, standard deviations, skewness, and kurtosis. Although the authors previously stated significant departures from normality, the vast majority of items fell within acceptable thresholds (skewness between −2 and 2; kurtosis between −7 and 7), suggesting that item distributions were approximately normal.

### 3.2. Internal Structure: Confirmatory Factor Analysis (Sample A)

The initial confirmatory factor analysis (CFA) conducted on Sample A (n = 413) was based on the two-factor model proposed by Guo et al. (2017). However, the model showed poor fit: Satorra–Bentler χ^2^ (522) = 344.17, *p* < 0.0001; RMSEA = 0.095 (90% CI: 0.92–0.992); SRMR = 0.15; CFI = 0.54; NFI = 0.48; NNFI = 0.51. Given these suboptimal indices, exploratory factor analysis (EFA) was deemed necessary to examine the underlying structure in greater depth.

### 3.3. Exploratory Factor Analysis (Sample A)

An EFA using unweighted least squares (ULS) with varimax rotation extracted two factors explaining 20.59% and 11.62% of the total variance:

Factor 1 (Compulsive Behaviors) included items such as washing, arranging, checking, grooming, planning, and social networking.

Factor 2 (Impulsive Behaviors) encompassed behaviors like substance use, aggression, self-injury, tattooing, gambling, and lying.

### 3.4. Confirmatory Factor Analysis (Sample B)

The CFA on Sample B (n = 278) tested the structure derived from the EFA. The model showed good fit: Satorra–Bentler χ^2^ (526) = 825.26, *p* < 0.0001; RMSEA = 0.048 (95% CI: 0.041–0.054); CFI = 0.92; NNFI = 0.91; SRMR = 0.06. All item loadings were significant, except for item 30 (social networking), which was retained for theoretical reasons (see Table 3).

### 3.5. Reliability and Item Analysis

Internal consistency was satisfactory for both subscales:

- Compulsive Behaviors: ω = 0.80, α = 0.79
- Impulsive Behaviors: ω = 0.79, α = 0.80

Corrected item-total correlations ranged from 0.18 to 0.52 for Factor 1, and from 0.28 to 0.59 for Factor 2, indicating adequate internal homogeneity.

### 3.6. Convergent Validity

Spearman correlations supported the convergent validity of the ICB:

- Compulsive Behaviors correlated negatively with Lack of Premeditation (−0.230) and Lack of Perseverance (−0.221), and positively with Sensation Seeking (0.178), and two OBQ-44 subscales (Responsibility/Threat Estimation, Importance/Control of Thoughts).
- Impulsive Behaviors showed strong positive correlations with Negative Urgency (0.521), Positive Urgency (0.428), Sensation Seeking (0.391), and all OBQ-44 and EuropASI dimensions (except Family/Social Relationships).
- Regarding the SCL-90-R, Factor 2 was significantly associated with all nine clinical dimensions, while Factor 1 showed small positive correlations only with Obsessive–Compulsive and Depression subscales (see Table 4 and Table 5).

## 4. Discussion

This study evaluated the psychometric properties of the Spanish version of the Impulsive-Compulsive Behaviours Checklist (ICB) in a large sample of incarcerated men. As anticipated, impulsivity and compulsivity emerged as distinct but related constructs, in line with transdiagnostic models of behavioral dysregulation (López-Torrecillas, 2025; Robbins et al., 2024). The internal consistency, factorial structure, and convergent validity of the ICB were generally supported, extending its applicability to forensic populations.

The initial CFA did not replicate the original two-factor structure proposed by Guo et al. (2017), likely due to sample differences (community vs. prison). However, the EFA revealed a conceptually coherent solution that was subsequently confirmed in a second sample, indicating a stable two-factor model: Compulsive Behaviors (Factor 1) and Impulsive Behaviors (Factor 2). These findings align with theoretical distinctions between internally driven, rule-bound compulsions and externally oriented, disinhibited impulsive acts.

Correlational analyses further supported the scale’s validity. Compulsive Behaviors were weakly associated with psychopathological symptoms, suggesting a more rigid but potentially adaptive coping style in institutional settings. In contrast, Impulsive Behaviors showed strong associations with affect-driven impulsivity (UPPS-P), dysfunctional beliefs (OBQ-44), addiction severity (EuropASI), and clinical symptoms (SCL-90-R), highlighting their relevance to risk management and treatment needs in correctional environments.

These results suggest that the ICB can effectively differentiate between compulsive and impulsive profiles, offering practical value for mental health screening and individualized rehabilitation plans in prisons. The low correlation between Factor 1 and clinical variables does not negate its clinical relevance but may reflect a more subtle form of dysregulation characterized by cognitive rigidity rather than emotional dysregulation. (Appendix A and Appendix B show the definitive scale.)

## 5. Limitations and Future Directions

This study has several limitations. First, the sample was restricted to male inmates, limiting generalizability to female or non-incarcerated populations. This restriction was based on two main reasons: (1) the study assessed offenses such as gender-based violence, typically defined as male-to-female aggression; and (2) the prison population is predominantly male, with five times more men than women. Second, the lack of a control group prevents direct comparison with community norms. Third, no clinical diagnoses were included, precluding examination of discriminant validity across psychiatric subtypes.

Future research should explore the discriminative power of individual ICB items in differentiating impulsivity and compulsivity profiles and examine its relevance to specific diagnoses such as antisocial or obsessive–compulsive personality disorder. Studies involving broader samples—including women—and applying the ICB alongside symptom-based scales and longitudinal follow-up will be essential for establishing its predictive validity and clinical utility. Additionally, its use as a screening tool and treatment outcome measure in forensic populations warrants further investigation.

## 6. Conclusions

The Spanish version of the Impulsive-Compulsive Behaviours Checklist (ICB) demonstrates good psychometric properties in a prison population. The instrument offers a valid and reliable means of assessing impulsivity and compulsivity as distinct behavioral dimensions in forensic contexts. Its use could inform risk assessment, clinical diagnosis, and tailored interventions. Future research should explore its predictive validity and include behavioral or ecological measures (e.g., vignettes, real-life scenarios) to enhance the instrument’s applicability.

## Figures and Tables

**Table 1:** Baseline demographic and crime behavior characteristics of the participants (N = 700) and refused (N = 50).

Variables Scores	
Education (N = 700)	
Without Primary	115
Primary	325
Secondary	237
Degree	23
Education (N = 50)	
Without Primary	2
Primary	31
Secondary	7
Degree	0
Marital status (N = 700)	
Single	332
Married	135
Divorced	99
Widower	6
Lived with their partner	128
Marital status (N = 50)	
Single	25
Married	9
Divorced	5
Widower	1
Lived with their partner	10
Nationality (N = 700)	
Spain	661
Other European	7
South America	17
Africa	15
Nationality (N = 50)	
Spain	46
Other European	4
South America	0
Africa	0
Offenses (N = 700)	
Against life and integrity	67
Against freedom	52
Against property/treasury	343
Against public health	112
Gender violence	116
Offenses (N = 50)	
Against life and integrity	5
Against freedom	3
Against property/treasury	28
Against public health	3
Gender violence	11
Months of the prison sentence mean (SD)	81.51 (79.77)
Months of the prison sentence (Range)	(3–680)

**Table 2:** Descriptive statistics for item scores: Mean (M), Standard deviation (SD), Skewness, and Kurtosis.

Item	M	SD	Skewness	Kurtosis
1.Washing	3.70	0.65	−1.725	4.197
2. Smoking	3.00	1.14	−0.467	−0.930
3. Feel compelled to collect free things (books, journals, sample items when shopping) or saving something you know you will never use	1.56	0.80	1.740	3.556
4. Being overly cautious with money	2.24	1.01	0.532	−0.606
5. (Re) Arranging/Ordering	2.90	0.96	−0.089	−0.895
6. Shopping	2.76	0.91	0.005	−0.717
7. List making	2.04	0.98	0.675	−0.519
8. Counting (e.g., money, tiles)	2.34	1.06	0.476	−0.674
9. Grooming	3.69	0.67	−1.566	3.009
10. Idiosyncratic routines (performing a very personalized sequence of actions)	2.80	0.98	−0.325	−0.748
11. Repeating actions (performing actions over and over again)	2.14	0.98	0.672	−0.194
12. Exercising	2.74	0.99	0.033	−0.948
13. Betting/Gambling	1.61	0.79	1.356	1.651
14. Hair picking	2.00	0.89	0.720	−0.016
15. Lying	1.89	0.61	1.140	4.733
16. Sexual activities/behaviours	2.47	0.93	0.184	−0.751
17. Calorie counting	1.37	0.69	1.964	3.339
18. Alcohol consumption	2.11	0.92	0.919	0.603
19. Planning (e.g., over organizing	2.08	0.90	0.731	0.307
20. Illicit drug use	2.28	1.14	0.618	−0.465
21. Cleaning too much	2.34	0.96	0.481	−0.283
22. Verbal aggression	1.75	0.66	0.632	0.591
23. Violence towards objects/properties	1.37	0.64	1.827	3.348
24. Swearing	1.81	0.71	0.784	0.934
25. Checking (e.g., locks, light switches)	1.95	0.95	0.887	0.085
26. Checking (e.g., yourself in the mirror)	2.32	0.92	0.555	−0.189
27. Speeding when driving	2.13	0.97	0.692	−0.145
28. Medication use	2.00	0.92	0.925	0.446
29. Physical aggression	1.92	0.82	0.650	0.099
30. Social networking (e.g., Facebook, Twitter, Google+, Myspace)	2.17	1.08	0.514	−0.839
31. Applying rules	2.64	0.93	0.129	−0.933
32. Purposeful self-injury (i.e., non-accidental)	1.24	0.56	2.838	9.939
33. Re-writing/re-reading	1.92	0.85	0.929	0.737
34. Tattooing	1.95	0.95	1.014	0.529

**Table 3:** Factorial structure. EFA and standardized solution for CFA.

	EFA		CFA	
Item	F1	F2	F1	F2
1.Washing	0.47		0.32	
3. Feel compelled to collect free things (books, journals, sample items when shopping) or saving something you know you will never use	0.42		0.19	
4. Being overly cautious with money	0.37		0.38	
5. (re) Arranging/Ordering	0.60		0.58	
6. Shopping	0.45		0.47	
7. List making	0.54		0.49	
8. Counting (e.g., money, tiles)	0.47		0.51	
9. Grooming	0.63		0.41	
10. Idiosyncratic routines (performing a very personalized sequence of actions)	0.53		0.47	
11. Repeating actions (performing actions over and over again)	0.47		0.45	
12. Exercising	0.45		0.18	
14. Hair picking	0.31		0.29	
17. Calorie counting	0.40		0.22	
19. Planning (e.g., over organizing)	0.53		0.50	
21. Cleaning too much	0.62		0.50	
25. Checking (e.g., locks, light switches)	0.40		0.47	
26. Checking (e.g., yourself in the mirror)	0.50		0.58	
30. Social networking (e.g., Facebook, Twitter, Google+, Myspace)	0.35		0.21	
31. Applying rules	0.36		0.16	
33. Re-writing/re-reading	0.38		0.33	
2. Smoking		0.44		0.28
13. Betting/Gambling		0.53		0.50
15. Lying		0.50		0.51
16. Sexual activities/behaviours		0.32		0.31
18. Alcohol consumption		0.50		0.36
20. Illicit drug use		0.70		0.54
22. Verbal aggression		0.70		0.58
23. Violence towards objects/properties		0.77		0.77
24. Swearing		0.54		0.54
27. Speeding when driving		0.60		0.60
28. Medication use		0.51		0.37
29. Physical aggression		0.31		0.33
32. Purposeful self-injury (i.e., non-accidental)		0.63		0.55
34. Tattooing		0.39		0.41

**Table 4:** Spearman’s correlations between factor 1 and 2 of ICB and the dimensions of UPPS P, OBQ-44 and EuropASI.

	ICB Factor 1	ICB Factor 2		ICB Factor 1	ICB Factor 2
UPPS-P			EuropASI		
Lack of Premeditation	−0.230 **	0.204 **	Medical status	0.087	0.146 *
Negative Urgency	0.067	0.521 **	Employment/support	0.018	0.195 **
Lack of Perseverance	−0.221 **	0.095	Alcohol use	0.008	0.437 **
Sensation Seeking	0.178 **	0.391 **	Drug use	0.018	0.590 **
Positive Urgency	0.051	0.428 **	Family/social relationships	−0.003	0.040
OBQ-44 Responsibility and threat estimation	0.205 **	0.226 *	EuropASI Total	0.082	0.617 **
Importance and control of thoughts	0.289 **	0.134 *			
Perfectionism/certainty	0.081	0.140 *			

*Note.* ICB Checklist = Impulsive-Compulsive Behaviours Checklist; OBQ-44 = Obsessive Beliefs Questionnaire-44; UPPS-P = Positive Urgency Impulsive; EuropASI = European Addiction Severity Index. ** *p* < 0.001, * *p* < 0.005.

**Table 5:** Spearman’s correlations between factors 1 and 2 of ICB and dimensions of SCL-90.

	ICB Factor 1	ICB Factor 2
SCL-90		
Somatization	0.088	0.152 **
Obsessive–compulsive	0.194 **	0.298 **
Interpersonal sensitivity	0.086	0.216 **
Depression	0.159 **	0.195 **
Anxiety	0.107	0.324 **
Hostility	0.098	0.293 **
Phobic anxiety	0.063	0.164 **
Paranoid ideation	0.083	0.156 **
Psychoticism	0.069	0.278 **

*Note*. SCL-90 = The Symptom Checklist-90. ** *p* < 0.001.

## Data Availability

The data presented in this study are available upon reasonable request from the corresponding author. The data are not publicly available due to privacy and ethical restrictions.

## Appendix A. Impulsive-Compulsive Behaviours Checklist (ICB)

This list consists of several behaviours that we all engage in from time to time. It can be challenging to be honest about your level of involvement in these behaviours and therefore we emphasize that all information here will be confidential. You will not be judged in any way based on your answers and we encourage you to fill in the items on this list honestly and accurately. When considering your responses, please do not include issues that are caused by medical conditions (e.g., diabetes, erectile dysfunction).

Please answer the questions below for every behaviour on the list by selecting the appropriate response on the scale ranging from ‘Not at all’ to ‘All the time’. Please answer each question as it applies to you over the last 12 months.

Do YOU and/or OTHERS think you have an issue/problem with any of the following behaviours?

1 = Never; 2 = Sometimes; 3 = Often; 4 = Always.

	**1**	**2**	**3**	**4**
1. Washing				
2. Smoking				
3. Feel compelled to collect free things (books, journals, sample items when shopping) or saving something you know you will never use				
4. Being overly cautious with money				
5. (re) Arranging/Ordering				
6. Shopping				
7. List making				
8. Counting (e.g., money, tiles)				
9. Grooming				
10. Idiosyncratic routines (performing a very personalized sequence of actions)				
11. Repeating actions (performing actions over and over again)				
12. Exercising				
13. Betting/Gambling				
14. Hair picking				
15. Lying				
16. Sexual activities/behaviours				
17. Calorie counting				
18. Alcohol consumption				
19. Planning (e.g., over organizing				
20. Illicit drug use				
21. Cleaning too much				
22. Verbal aggression				
23. Violence towards objects/properties				
24. Swearing				
25. Checking (e.g., locks, light switches)				
26. Checking (e.g., yourself in the mirror)				
27. Speeding when driving				
28. Medication use				
29. Physical aggression				
30. Social networking (e.g., Facebook, Twitter, Google+, Myspace)				
31. Applying rules				
32. Purposeful self-injury (i.e., non-accidental)				
33. Re-writing/re-reading				
34. Tattooing				

## Appendix B. Listado de Comportamientos Compulsivos-Impulsivos (ICB)

A continuación le presentamos una lista de comportamientos que todos podemos realizar de vez en cuando. Lee cada frase y decide en qué grado te describe. No vas a ser juzgado/a por tus respuestas. No hay respuestas correctas o erróneas. Probablemente estarás de acuerdo con algunas frases y en desacuerdo con otras. Por favor, indica tus comportamientos y sentimientos personales sobre cada frase, marcando con una cruz lo que mejor describa tu conducta o sentimiento. Se muy sincero/a e intenta describirte cómo eres realmente es y no como te gustaría ser.

1 = Nunca; 2 = Algunas veces; 3 = A menudo; 4 = Siempre.

	**1**	**2**	**3**	**4**
1. Lavarme				
2. Fumar				
3. Coleccionar artículos gratuitos (libros, revistas, muestras de regalo) o guardar algo que sabes que nunca vas a utilizar				
4. Ser excesivamente prudente con el dinero				
5. Reorganizar y ordenar				
6. Comprar				
7. Hacer listas de tareas				
8. Contar (dinero, fichas, piezas, etc.)				
9. Asearme				
10. Rutinas personales				
11. Acciones repetitivas (hacer una y otra vez la misma actividad)				
12. Hacer ejercicio				
13. Hacer apuestas				
14. Tocarme o arrancarme el pelo				
15. Mentir				
16. Realizar comportamientos/actividades sexuales				
17. Contar calorías				
18. Consumir alcohol				
19. Planificar u organizar demasiado				
20. Consumir drogas ilegales				
21. Limpiar demasiado				
22. Realizar agresiones verbales				
23. Realizar violencia hacia objetos de valor				
24. Realizar juramentos				
25. Realizar comprobaciones (cerraduras, interruptores de luz, etc.)				
26. Realizar comprobaciones (ejemplo, mirarme en el espejo)				
27. Conducir a alta velocidad				
28. Usar medicamentos				
29. Agredir físicamente				
30. Usar redes sociales por ejemplo Facebook, Twitter, Google+, Myspace				
31. Regirme por las normas				
32. Autolesionarme conscientemente (no de manera accidental)				
33. Reescribir y releer				
34. Hacerme tatuajes				
